# Depression, C-Reactive Protein, and the Risk of Mortality During Middle and Late Adulthood

**DOI:** 10.3390/epidemiologia7050133

**Published:** 2026-09-26

**Authors:** Srikanta Banerjee, Jagdish Khubchandani, May A. Beydoun, Stanley Nkemjika, Kavita Batra

**Affiliations:** 1 College of Health Sciences, Walden University, Minneapolis, MN 55401, USA; srikanta.banerjee2@mail.waldenu.edu; 2 College of Health, Education, and Social Transformation, New Mexico State University, Las Cruces, NM 88003, USA; 3 Laboratory of Epidemiology and Population Sciences, National Institute on Aging, National Institute on Aging/NIH/IRP, Baltimore, MD 21224, USA; baydounm@mail.nih.gov; 4 Department of Psychiatry and Human Behavior, Thomas Jefferson University, Philadelphia, PA 19107, USA; 5 Department of Medical Education and Office of Research, Kirk Kerkorian School of Medicine at UNLV, University of Nevada, Las Vegas, NV 89102, USA; kavita.batra@unlv.edu

**Keywords:** depression, C-reactive protein, inflammation, mortality

## Abstract

**Background and Purpose:** Mental illnesses, including depression, are major contributors to disability and health loss globally. Research has increasingly focused on identifying biochemical markers, such as C-reactive protein (CRP), to better understand the link between systemic inflammation and psychiatric disorders. However, the long-term health impacts of their coexistence are underexplored. This study aimed to investigate the relationship between depression, elevated CRP levels, and mortality risk among community-dwelling older adults in the United States. **Methods:** Data from the National Health and Nutrition Examination Survey (NHANES) from 2005–2010 were utilized, linked with mortality data up to December 2019. The sample included 4606 adults aged 50 years and older. Depression was assessed through self-reported measures, and elevated CRP was defined as serum CRP ≥ 2 mg/L. Participants were categorized into four groups: depression only, elevated CRP only, both conditions, or neither. Cox regression models were employed to estimate hazard ratios (HRs) for all-cause and cardiovascular mortality, adjusting for sociodemographic and health-related covariates. **Results:** Individuals with both depression and elevated CRP had the highest risk of all-cause mortality (HR = 3.56, 95% CI = 1.41–8.97) and cardiovascular mortality (HR = 14.42, 95% CI = 4.29–21.06) compared to those with neither condition. Elevated CRP alone and depression alone were also independently associated with increased mortality risk. Predictors such as age, poverty, smoking, obesity, diabetes, and cardiovascular history significantly influenced these associations. **Conclusion:** The coexistence of depression and systemic inflammation markedly elevates the risk of mortality. These findings highlight the need for integrated clinical strategies targeting both mental health and inflammation to mitigate mortality risks in vulnerable populations.

## 1. Introduction

Mental illnesses are a leading cause of disability and health loss globally. It is estimated that more than a tenth of the global population lives with a mental illness, equating to nearly a billion people [1,2,3]. In 2019, mental illnesses accounted for more than 30 million years of life with disability (nearly 20% of all the years lived with disability from all causes worldwide). The COVID-19 pandemic further increased the prevalence of mental illnesses worldwide and has also acted as a catalyst for a flurry of research on mental health and psychiatric illnesses [2,3,4,5]. Interestingly, within the past few decades, extensive research on biochemical and genetic markers in both human and animal models has been conducted to understand common mental illnesses (e.g., depression and anxiety). To comprehensively elucidate the molecular mechanisms underlying psychiatric disorders, it has been suggested that biomarkers should be assessed at multiple biological levels, a methodology popularly known as the “omics” approach [6,7,8,9]. Despite significant progress, the search for definitive and distinct biomarkers for psychiatric disorders remains ongoing. For instance, numerous recent studies have reported on the patterns of multiple immune, metabolic, inflammatory, genetic, and endocrine biomarkers among individuals with depression (e.g., interleukins, growth factors, telomere length, leptin, neurotransmitter receptors, etc.) [7,8,9,10,11,12].

Of all the biomarkers for depression, C-reactive protein (CRP) is among the most widely appraised and investigated markers via a variety of studies. Many of these studies report the presence of chronic low-grade inflammation as measured by CRP levels among individuals with depression in both clinical and community samples [12,13,14,15,16,17,18,19,20,21,22]. The major ambiguity in these studies relates to whether CRP levels predict the risk of depression or if depressive disorders precede the elevation of CRP. For example, a study using the Irish Longitudinal Study on Ageing (TILDA) reported that after a follow-up of 4 years, higher serum CRP levels were significantly associated with a risk of depression among those with comorbidities [13]. Furthermore, an analysis of the Copenhagen General Population and City Heart studies found that elevated CRP at baseline was associated with a higher risk of psychological distress, antidepressant usage, or hospitalization with depression [14]. These studies and others have also reported that elevated CRP may lead to development, progression, symptom severity, and even specific depressive psychopathology [13,14,15,16]. In contrast, an analysis of the data on 13,775 older adults from the Health and Retirement Study in the U.S. found that depressive symptoms predicted increasing inflammation (as measured from CRP levels and only in men) [17]. Another study of 2544 healthy participants in the CARDIA study found that depressive symptoms at baseline predicted CRP levels after 5 years independent of sociodemographic characteristics, lifestyle behaviors, medical and biological risk factors, and baseline CRP [18].

Recently published studies and meta-analyses have suggested that there could be a bidirectional relationship between depression and CRP [18,19,20,21]. For example, the latest analysis of the Chinese Health and Retirement Longitudinal Study on more than 11,000 adults found an association between sustained CRP elevations over two successive determinations with subsequent risk of depressive symptoms and also between repeated episodes of depression and later risk of elevated CRP [19]. Similarly, the largest meta-analysis to date on this topic analyzed 25 studies with elevated CRP and subsequent risk of depression (17 studies with depression at baseline and later elevation of CRP from this review suggest a bidirectional relationship) [20]. Researchers have also suggested that it is difficult to establish the directionality of the relationship between depression and CRP due to covariates (e.g., genotype, diet, lifestyles, health conditions, and sociodemographic characteristics) [20,21,22]. Beyond the direction of the relationship between depression and CRP, the long-term effect of coexisting depression and elevated CRP has not been established. Thus, the purpose of this study was to assess the risk of mortality among a community-dwelling sample of adult Americans based on CRP levels and depression after accounting for multiple sociodemographic and health-related characteristics.

## 2. Methods

### 2.1. Participants and Procedures

For this study, we utilized the National Health and Nutrition Examination Survey (NHANES) data from 2005–2010 by merging data from individual NHANES cycles (i.e., 2005–2006, 2007–2008, 2009–2010) [12,22,23,24,25,26,27]. NHANES is a nationally representative, cross-sectional survey administered by the National Center for Health Statistics (NCHS) that uses a complex, multistage probability sampling design to assess the health and nutritional status of the U.S. civilian non-institutionalized population. Participants are recruited across the entire United States, and data collection comprises two components: an in-home interview and a physical examination conducted at a mobile examination center. For this analysis, eligibility required participants to be 50 years of age or older at the time of their NHANES examination. Data were collected once per NHANES cycle for each participant (i.e., no repeated measures within cycles); however, mortality was subsequently tracked from the examination date through December 2019 using linked National Death Index (NDI) records. Depression in NHANES was assessed using the Patient Health Questionnaire-9 (PHQ-9), a validated 9-item self-report instrument that asks participants to rate the frequency of depressive symptoms over the previous two weeks. Participants scoring 10 or higher on the PHQ-9 were classified as having clinically significant depression, consistent with established cutoffs for moderate-to-severe depression. This assessment was conducted at the time of the NHANES examination. Data collection for this national survey comprises two components (i.e., interview and physical exam). The survey interview includes questions about medical conditions, lifestyles, and sociodemographic characteristics. The physical exam component includes medical, physiological, and laboratory measurements (e.g., CRP) [12,22,24]. We linked this NHANES data on adults 50 years of age or older with mortality data up to December 2019 by utilizing the death certificate records from the National Death Index (NDI) [23,25,27]. Blood samples of study participants were collected in NHANES, and for CRP, latex-enhanced nephelometry with particle-enhanced assays were used for quantification. The primary standard used for processing was organized by Behring Diagnostics and standardized against WHO reference material. A cutoff level of ≥2 mg/L was considered elevated CRP in study participants [22,25,26]. Our analysis examined participant characteristics including age, gender, education level, race/ethnicity, and health insurance status. Family income to poverty ratio (FIPR) was utilized to assess household income. The prevalence of obesity was calculated using participants’ height and weight measurements to compute BMI, which was dichotomized for regression analysis. For regression analysis, BMI was categorized using standard WHO criteria: normal weight (BMI 18.5–24.9 kg/m^2^), overweight (25.0–29.9 kg/m^2^), obese (30.0–39.9 kg/m^2^), and morbidly obese (≥40.0 kg/m^2^), and then dichotomized as non-obese (BMI < 30) versus obese (BMI ≥ 30) in regression models. Regarding the CRP threshold, a cutoff of ≥2 mg/L was chosen as it represents a commonly used clinical threshold indicative of low-grade systemic inflammation that has been associated with increased cardiovascular and all-cause mortality risk in prior population-based studies. This threshold is consistent with the American Heart Association and CDC guidelines that 1–3 mg/L as average risk. Our use of ≥2 mg/L captures individuals with at least moderate inflammatory burden, and also, this cutoff has been used in multiple prior studies from this database [20,21,22,25,26]. Marital status was categorized into two groups for regression analysis: those who are married or cohabiting versus those who are widowed, divorced, separated, or never married. Diabetes and smoking prevalence were based on self-reported data from participants. Additionally, a history of cardiovascular disease was identified through self-reported diagnoses of coronary heart disease, hypertension, stroke, or myocardial infarction. For chronic kidney disease (CKD), the glomerular filtration rate was calculated using the Cockcroft–Gault equation. The prevalence of chronic obstructive pulmonary disease (COPD) was determined by combining self-reported diagnoses of emphysema or chronic bronchitis. Comprehensive details regarding the NHANES data analysis and methodologies have been extensively documented in studies published elsewhere [22,23,24,25]. All study protocols and procedures received approval from the NCHS Research Ethics Review Board before data collection.

### 2.2. Data Analysis

The sociodemographic and health-related characteristics of the study participants were analyzed using descriptive statistics (e.g., frequencies and percentages with 95% confidence intervals). Subsequently, Rao-Scott Chi-square tests were utilized to assess differences between those with and without depression for sociodemographic and health-related characteristics. Prior to fitting the Cox regression models, we initially incorporated tests based on Schoenfeld residuals to assess the proportional hazards and also checked for assumption independent censoring. Multivariate analysis was performed via the construction of Cox regression models to examine differences in mortality among people with depression (based on whether or not they had elevated CRP). The analysis involved four groups: individuals with only depression, those with only elevated CRP, participants with both depression and elevated CRP, and a reference group with neither condition, which was used for mortality evaluation (this last group served as the reference group). These regression models were adjusted for health-related (e.g., comorbidities) and sociodemographic characteristics. Individuals who did not have data on CRP or depression were excluded from this analysis. Statistical significance was assumed to be at an alpha level of *p* < 0.05.

## 3. Results

A total of 4606 individuals were included in our study sample with an average age of 65.2 years (SE = 0.27), and the majority were females (51.3%), married (51.7%), and white (78.3%). Also, nearly a fifth were living in poverty, currently smoking, or had diabetes, CVD, or CKD. When the total population was stratified by depression status, those who had depression were significantly more likely to be female, younger, poorer, with lower education, racial minorities, smokers, obese, and to have diabetes, CVD, or CKD (Table 1).

For overall mortality, the adjusted hazard ratio (HR) for those with depression compared to those without depression was 2.17 (95% CI = 1.69–2.78), irrespective of the CRP levels. Among the various groups, after adjustment for sociodemographic and health variables, individuals with depression but without elevated CRP had a significantly higher likelihood of mortality (HR = 1.32, 95% CI = 1.05–1.66). Also, among individuals with elevated CRP but without depression, the risk of mortality was significantly higher (HR = 1.99, 95% CI = 1.52–2.61). Among those with both depression and elevated CRP, the risk of mortality was statistically significantly elevated and the highest across all groups (HR = 3.56, 95% CI = 1.41–8.97). Across all these comparison groups, age, poverty, smoking, obesity, diabetes, CKD, and CVD were significant covariates for the relationship between depression, CRP, and mortality (Table 2).

For cardiovascular disease mortality, the risk of mortality for those with depression compared to those without depression was 3.62 (95% CI = 2.44–5.37)^,^ irrespective of CRP levels. Among the various groups, after adjustment for sociodemographic and health variables, individuals with depression but without elevated CRP did not have a significantly higher likelihood of CVD mortality (HR = 1.13, 95%CI = 0.69–1.86). In contrast, among individuals with elevated CRP but without depression, the risk of cardiovascular mortality was significantly higher (HR = 2.83, 95% CI = 1.85–4.32). Among those with both depression and elevated CRP, the risk of cardiovascular mortality was statistically significantly elevated and the highest across all groups (HR = 14.42, 95% CI = 4.29–21.06). Across all these comparison groups, age, diabetes, and CVD history were significant covariates for the relationship between depression, CRP, and CVD mortality (Table 3).

## 4. Discussion

In this national study of American adults, we found a notable association between depression, elevated CRP, and an increased risk of mortality. Our findings are consistent with earlier studies that emphasized the significance of chronic inflammation in both physical and mental health outcomes. CRP, which serves as an indicator of systemic inflammation, is frequently elevated in various medical conditions, including cardiovascular diseases, autoimmune disorders, and infections [9,10,11,12]. The connection between CRP and depression has gained recognition, with research showing that individuals suffering from depression tend to have higher CRP levels compared to healthy individuals [14,15,16]. This inflammatory condition may play a role in the pathophysiology of depression, as inflammatory cytokines are known to affect neurotransmitter metabolism, brain function, and mood regulation. Notably, our finding that depression alone (without elevated CRP) was not significantly associated with elevated cardiovascular disease mortality (HR = 1.13, 95%CI = 0.69–1.86) warrants discussion, as this differs from some prior studies linking depression independently to CVD outcomes. One possible explanation is that in this community-dwelling older adult sample, the inflammatory burden captured by elevated CRP may be a necessary co-factor for the depression-CVD mortality pathway to become statistically detectable. This observation raises the question of whether CRP mediates or moderates the relationship between depression and CVD mortality. While formal mediation analysis was beyond the scope of this study, our group-comparison approach is consistent with prior findings suggesting that CRP may serve as a critical biological mediator linking depression to cardiovascular risk—a pattern observed in Mendelian randomization analyses as well [28,29,30]. Future studies with larger samples should formally test mediation to disentangle these pathways.

A major finding of this study was that the simultaneous occurrence of depression and elevated CRP levels was linked to an increased risk of mortality. The pathways underlying this relationship could be multifactorial (e.g., immune, vascular, inflammatory) [28,29,30,31]. For example, studies have found that chronic low-grade inflammation (e.g., elevated CRP) levels may serve as a precursor for chronic conditions (e.g., cancer and cardiovascular disease), posing higher long-term mortality risks [8,9,10,11,12]. The combined impact of depression and inflammation may intensify the physiological strain on the body, thereby increasing vulnerability to negative health events. Furthermore, depression is linked with dysregulation of the hypothalamic–pituitary–adrenal (HPA) axis, heightened activity of the sympathetic nervous system, and changes in immune responses, all of which can lead to an inflammatory state [17,18,19,20]. Such chronic inflammation can lead to the early onset and progression of various conditions, such as cardiometabolic disorders and neurodegenerative disorders, which are all connected to a higher risk of mortality [28,29,30,31,32]. The interplay between depression and increased CRP levels may indicate a more extensive pathway that heightens the risk of early death for individuals.

The study findings have major implications for practice and prevention. Our results highlight the necessity for comprehensive treatment strategies for those experiencing both depression and elevated CRP levels. Addressing both the psychological and inflammatory components of their health could result in better outcomes. Hence, there is a need for integrating inflammatory markers into mental health assessment frameworks. Traditionally, depression has been approached from a psychosocial and neurochemical perspective; however, our findings reinforce the emerging paradigm that systemic inflammation plays a significant role in the pathophysiology of depressive disorders. Additionally, depression not only diminishes quality of life but, when coupled with elevated CRP, may signal an elevated risk for cardiovascular, metabolic, and other inflammation-related diseases. Interventions like antidepressant medications, lifestyle changes (such as exercise and dietary adjustments), and anti-inflammatory treatments may effectively alleviate depressive symptoms and inflammation, thus reducing the heightened mortality risk faced by this group. Chu and colleagues (2021) suggest multiple prevention strategies (e.g., primary vs. secondary prevention) for various groups (e.g., high-risk vs. at-risk) with depression and inflammation [33]. Primary prevention strategies include lifestyle alterations such as the promotion of healthier diets, physical activity, and stress management among individuals with depression or inflammation [33,34]. Secondary prevention strategies include early detection of inflammation, preventing recurrence of depression episodes by early diagnosis and management, and use of adjunct anti-inflammatory treatments that are more effective than using antidepressants alone [34,35,36]. Public health initiatives should also support longitudinal assessments to further elucidate causal pathways and to develop targeted, inflammation-focused interventions for at-risk populations.

This study has several key limitations. Firstly, cross-sectional design restricts our ability to determine cause-and-effect relationships between depression, CRP levels, and mortality. To gain a clearer understanding of how these factors interact over time and their overall impact on health, longitudinal studies are necessary. Secondly, the research focused solely on CRP as a marker of inflammation, but it’s crucial to recognize that inflammation is a complex process influenced by multiple factors. Exploring other biomarkers or indicators of systemic inflammation could shed light on the mechanisms behind the observed connections. Another limitation is that the very high CVD mortality hazard ratios reported for participants with both depression and elevated CRP should be interpreted more cautiously because the confidence intervals are relatively wide, suggesting that these estimates may be less precise (e.g., HR is 3.56 (95% CI 1.41–8.97) for all-cause mortality and 14.42 (95% CI 4.29–21.06) for cardiovascular mortality). Also, if the combined group is small, the reliability of this finding may be limited by low power. Nevertheless, in our study, even after subgroup analysis, the fairly large sample size still leads to adequate power and avoids data imbalance. In addition, the potential for over-adjustment or multicollinearity between CRP and these comorbidities may meaningfully affect the reported hazard ratios. However, in medical sciences, multicollinearity is common, expected, and structurally inherent in part due to how social constructs are measured. Lastly, although we accounted for various confounding variables, there may still be unmeasured factors, such as socioeconomic status and healthcare access, that could affect the results.

Given the aforementioned limitations, future research should delve into the complex, bidirectional relationship between depression and systemic inflammation, with particular attention to longitudinal trajectories and underlying biological mechanisms. Studies should focus on longitudinal cohort designs that include repeated assessments of biomarkers and depression to better understand the temporal and possibly bidirectional relationship between systemic inflammation and depressive symptoms. The current study’s cross-sectional design does not allow us to determine whether elevated CRP occurs before, after, or simultaneously with depressive episodes over time. Also, to clarify the role of CRP, formal mediation and moderation analyses are essential, ideally utilizing Mendelian randomization or other quasi-causal methods. This will help ascertain whether CRP serves as a biological mediator, a moderator, or an independent pathway linking depression to cardiovascular and overall mortality. This is particularly important in light of the unexpected result that depression alone did not show a significant association with cardiovascular mortality in this sample. Multi-OMICS approaches—such as proteomics and metabolomics—are essential to identify mediating pathways and potential biomarkers, especially across distinct depression subtypes as well as various other mental disorders. Since CRP represents only one aspect of a complex inflammatory response, future research should include a wider array of immune, metabolic, and endocrine markers (such as interleukin-6, tumor necrosis factor-alpha, fibrinogen, and cortisol) along with multi-omics approaches—proteomic, metabolomic, and genomic—to uncover mediating pathways. Additionally, it is crucial to investigate whether different subtypes of depression (like atypical, melancholic, and treatment-resistant) exhibit varying inflammatory profiles and mortality risks. Integrated interventions that simultaneously address mental health and inflammatory processes should be developed and tested. Crucially, future studies must also account for disparities (e.g., socioeconomic) that may exacerbate both mental illness and mortality risk.

Interdisciplinary efforts are needed to clarify causal pathways and inform targeted strategies for prevention and treatment in vulnerable populations. Recognizing the interconnected nature of mental and physical health is crucial for improving patient outcomes and reducing mortality associated with chronic conditions and psychiatric illnesses. Also, considering the socioeconomic patterns of both depression and inflammation identified in this and previous research, it is imperative that future studies explicitly incorporate social determinants of health—such as poverty, education, healthcare access, and racial/ethnic disparities—as potential confounding variables, effect modifiers, or independent factors influencing the depression-inflammation-mortality pathway. This approach is essential to prevent misattributing biological causation to phenomena that may be significantly influenced by structural inequities. Furthermore, there is a pressing need for intervention trials to assess whether integrated treatment strategies that combine antidepressant medications, anti-inflammatory treatments, and lifestyle changes (including diet, physical activity, and stress management) can more effectively reduce mortality risk compared to single-modality treatments. Additionally, implementation research should investigate the practicality and clinical relevance of routinely incorporating CRP or other inflammatory markers into mental health assessment protocols for older adults, especially within primary care and geriatric contexts, where such integration could substantially enhance risk stratification and facilitate early intervention. Finally, this study focused on all-cause and cardiovascular mortality as primary outcomes. Whether depression and elevated CRP are associated with unnatural causes of death—including suicide and accidental mortality—remains an important unanswered question. Depression is a well-established risk factor for suicide, and while elevated inflammatory markers have been linked to suicidal ideation in some studies, the present analysis was not powered to examine cause-specific mortality beyond cardiovascular disease. Future studies should examine a broader spectrum of mortality causes, including suicide and injury-related deaths, in relation to depression-inflammation profiles. So, research should extend beyond general and cardiovascular mortality to explore cause-specific outcomes, such as suicide and injury-related deaths, given the well-documented connection between depression and suicide risk, as well as the emerging but under-researched associations between inflammatory markers and suicidal thoughts.

## 5. Conclusions

This study highlights the critical interplay between depression, systemic inflammation, and mortality risk among middle- and older-age adults. Findings demonstrate that both depression and elevated CRP independently increase the risk of all-cause and cardiovascular mortality. Notably, individuals with both depression and elevated CRP faced the highest risk, underscoring the compounded vulnerability associated with the coexistence of psychological distress and chronic inflammation. The results emphasize the necessity of integrated clinical approaches that address both mental health and physical health factors. Screening for depressive disorders and markers of systemic inflammation in clinical practice, particularly among older adults, could enable early interventions aimed at reducing long-term health risks. Multimodal strategies combining psychological care, anti-inflammatory treatments, and lifestyle modifications may provide the greatest benefit for individuals at risk.

## Figures and Tables

**Table 1:** Characteristics of Study Participants Stratified by Depression Symptoms.

Characteristics	Total Population (*n* = 4606)	Depression (+) (*n* = 649)	Depression (−) (*n* = 3957)
Age (Mean (SE) **	65.2 (0.27)	61.0 (0.46)	65.1 (0.32)
**Family Poverty–Income Ratio** (%) (Ref: PIR<1 = below poverty line) **	22.0 (19.8–24.2)	38.8 (32.9–44.7)	18.9 (16.7–21.2)
**Gender** (**Female**, %) **	51.3 (49.0–53.6)	56.5 (51.0–62.1)	50.4 (48.0–52.7)
**Health Insurance** (no, %) **	14.8 (13.0–16.6)	21.2 (17.3–25.0)	13.6 (11.8–15.5)
**Education Level** (%) *			
Some High School	33.3 (30.5–36.0)	45.1 (39.6–50.7)	31.1 (28.3–33.8)
High School Graduate	25.2 (23.3–27.0)	24.8 (20.2–29.4)	25.2 (23.3–27.2)
Some College & Beyond	41.6 (38.9–44.2)	30.1 (24.3–35.8)	43.7 (41.2–46.3)
**Ethnicity** (%) **			
Non-Hispanic White	78.3 (74.9–81.8)	72.5 (64.5–80.5)	78.5 (75.1–82.0)
Non-Hispanic Black	9.2 (7.5–11.2)	18.7 (12.3–25.2)	8.9 (7.2–10.2)
Hispanic	7.5 (5.7–9.8)	8.0 (4.9–12.9)	7.5 (5.7–9.7)
Other	5.0 (3.9–6.3)	0.8 (0.2–3.3)	5.1 (4.0–6.5)
**Marital Status** (%) *			
Married	51.7 (49.3–54.2)	40.8 (35.2–46.4)	53.8 (51.3–56.3)
Widowed	18.1 (16.7–19.6)	16.1 (13.5–18.8)	18.5 (17.0–20.0)
Divorced	15.9 (14.3–17.5)	21.7 (18.2–25.3)	14.8 (13.0–16.5)
Separated	3.6 (2.8–4.5)	7.0 (4.7–10.4)	2.9 (2.2–3.9)
Never Married	7.7 (6.6–8.9)	9.3 (7.2–12.0)	7.3 (6.2–8.6)
Living with Partner	3.0 (2.4–3.8)	5.1 (3.0–8.3)	2.7 (2.2–3.3)
**Elevated CRP,** (yes, %)	4.3 (3.6–5.0)	4.8 (3.0–7.7)	4.2 (3.5–5.0)
**Diabetes** (yes, %) **	22.6 (21.2–19.7)	30.0 (25.5–34.6)	21.2 (19.7–22.6)
**Smoking Status** (yes, %) **	20.3 (18.4–22.2)	33.5 (28.2–38.7)	17.8 (16.1–19.5)
**Cardiovascular Disease** (yes, %) **	24.0 (21.8–26.2)	32.6 (28.1–37.2)	22.4 (20.3–24.6)
**Chronic Kidney Disease** (yes, %) *	20.8 (19.0–22.7)	12.5 (8.9–16.0)	22.4 (20.2–24.6)
**Obesity** *			
Normal	24.6 (22.8–26.3)	23.2 (18.1–28.2)	24.8 (23.1–26.6)
Overweight	33.3 (31.8–34.9)	28.5 (24.0–33.0)	34.3 (32.6–35.9)
Obesity	33.8 (32.2–35.3)	36.4 (32.1–40.8)	33.3 (31.9–34.7)
Morbid Obesity	8.3 (7.3–9.4)	11.9 (8.9–15.0)	7.6 (6.7–8.7)
**Cardiovascular Deaths** (N, %)	380 (8.3%)	52 (8.6%)	328 (8.1%)
**All deaths** (N, %) *	1478 (32.1%)	221 (34.1%)	1257 (31.8%)

Note. * *p* < 0.05; ** *p* < 0.01. Numbers with 95 CI indicate 95% confidence intervals for proportions.

**Table 2:** Participants’ Risk of Overall Mortality Stratified by Depression and C-Reactive Protein Levels.

	Total Population HR (95%Ci) (Dep vs. Non-Dep) N = 1775	Elevated CRP & No Depression HR (95%Ci) N = 1629	Normal CRP & Depression HR (95%Ci) N = 1513	Elevated CRP & Depression History HR (95%Ci) N = 146
**Depression/CRP category**	2.17 (1.69–2.78) **	1.99 (1.52–2.61) **	1.32 (1.05–1.66) *	3.56 (1.41–8.97) **
**Age**	1.09 (1.08–1.12) **	1.10 (1.08–1.11) **	1.10 (1.08–1.11) **	1.07 (1.05–1.09) *
**Health Insurance** (ref: yes)	1.18 (0.86–1.62)	1.23 (0.85–1.78)	1.18 (0.84–1.67)	0.90 (0.42–1.94)
**Gender** (Ref: male)	0.68 (0.59–0.78) **	0.65 (0.56–0.76) **	0.68 (0.59–0.78) **	0.88 (0.52–1.49)
**Marital Status** (ref: living with a partner or married)	1.49 (1.25–1.77) **	1.54 (1.29–1.83) **	1.43 (1.20–1.69) **	1.02 (0.71–1.48)
**Education Level**				
Some college & beyond	Ref	Ref	Ref	Ref
<High School education	1.32 (1.12–1.55) *	1.31 (1.11–1.54) *	1.26 (1.05–1.51) *	1.00 (0.65–1.52)
High School Graduate	1.11 (0.96–1.28)	1.16 (1.00–1.35)	1.07 (0.92–1.24)	0.64 (0.36–1.13)
**Race/Ethnicity**				
Non-Hispanic White	Ref	Ref	Ref	Ref
Non-Hispanic Black	0.80 (0.66–0.97) *	0.82 (0.66–1.01)	0.81 (0.65–1.01) *	0.73 (0.42–1.26)
Hispanic	0.49 (0.37.0.66) **	0.58 (0.42–0.79) *	0.50 (0.37–0.67) **	0.22 (0.11–0.45) **
Other	0.96 (0.54–1.71)	0.91 (0.47–1.77)	0.96 (0.53–1.72)	1.02 (0.43–2.45)
**Family Poverty–Income Ratio** (Ref: PIR ≥ 1)	1.57 (1.29–1.92) **	1.47 (1.18–1.83) *	1.54 (1.24–1.91) *	1.72 (1.11–2.65) *
**Smoking Status** (ref: no)	1.54 (1.30–1.81) **	1.48 (1.21–1.82) *	1.52 (1.28–1.82) **	1.95 (1.22–3.14) *
**Chronic Kidney Disease** (ref: no)	1.41 (1.19–1.67) **	1.38 (1.15–1.65) *	1.39 (1.17–1.66) **	1.52 (1.05–2.21) *
**Obesity** (ref: no)	1.26 (1.07–1.48) *	1.20 (1.03–1.41) *	1.28 (1.08–1.52) *	2.12 (1.14–3.95) *
**Cardiovascular Disease** (ref: no)	1.86 (1.61–2.15) **	1.82 (1.56–2.14) **	1.81 (1.57–2.09) **	1.92 (1.30–2.81) *
**Diabetes** (ref: no)	1.82 (1.57–2.11) **	1.73 (1.46–2.04) **	1.89 (1.63–2.19) **	2.5 (1.69–3.69) **

Note. * *p* < 0.05; ** *p* < 0.01. HR (95% CI) indicates hazard ratios with 95% confidence intervals for the outcome (i.e., mortality). Ref indicates the reference group among each variable for comparison with other groups. For all group comparisons, individuals without depression or elevated CRP were treated as the control group. N indicates the number of individuals for whom the mortality and follow-up data were available.

**Table 3:** Participants’ Risk of CVD Mortality Stratified by Depression and C-Reactive Protein Levels.

	Total Population HR (95%Ci) (Dep vs. Non-Dep) N = 451	Elevated CRP & No Depression HR (95%Ci) N = 416	Normal CRP & Depression HR (95%Ci) N = 378	Elevated CRP & Depression History HR (95%Ci) N = 35
**Depression/CRP category**	3.62 (2.44–5.37) **	2.83 (1.85–4.32) *	1.13 (0.69–1.86)	14.42 (4.29–21.06) **
**Age**	1.11 (1.09–1.13) **	1.12 (1.09–1.15) *	1.12 (1.10–1.14) **	1.10 (1.04–1.16) *
**Health Insurance** (ref: yes)	0.56 (0.25–1.27)	0.63 (0.24–1.65)	0.57 (0.22–1.47)	0.25 (0.04–1.37)
Gender (Ref: male)	0.57 (0.41–0.79) *	0.51 (0.37–0.70) **	0.54 (0.37–0.77) **	0.82 (0.35–1.92)
**Marital Status** (ref: living with a partner or married)	1.61 (1.23–2.12) *	1.70 (1.27–2.27) *	1.60 (1.23–2.09) *	1.02 (0.41–2.58)
**Education Level**				
Some college & beyond	Ref	Ref	Ref	Ref
<High School Education	1.33 (1.05–1.69) *	1.41 (1.10–1.80) *	1.22 (0.91–1.64)	0.77 (0.26–1.38)
High School Graduate	1.14 (0.82–1.57)	1.23 (0.90–1.67)	1.03 (0.72–1.47)	0.59 (0.25–1.38)
**Race/Ethnicity**				
Non-Hispanic White	Ref	Ref	Ref	Ref
Non-Hispanic Black	0.83 (0.57–1.20)	0.92 (0.64–1.32)	0.84 (0.57–1.23)	0.56 (0.12–2.57)
Hispanic	0.58 (0.33–1.01)	0.59 (0.31–1.13)	0.62–(0.37–1.05)	0.53 (0.14–1.99)
Other	0.88 (0.30–2.63)	0.42 (0.12–1.46)	0.91 (0.31–2.71)	1.91 (0.17–21.4)
**Family Poverty–Income Ratio** (Ref: PIR ≥ 1)	1.22 (0.84–1.76)	1.08 (0.65–1.81)	1.19 (0.78–1.80)	1.40 (0.38–5.11)
**Smoking Status** (ref: no)	1.11 (0.84–1.46)	0.99 (0.73–1.36)	1.02 (0.75–1.39)	3.02 (1.14–8.01) *
**Chronic Kidney Disease** (ref: no)	1.39 (1.05–1.86) *	1.32 (0.99–1.77)	1.31 (0.94–1.82)	1.34 (0.48–3.73)
**Obesity** (ref: no)	1.04 (0.71–1.52)	0.88 (0.59–1.30)	1.04 (0.68–1.57)	4.73 (1.38–16.20) *
**Cardiovascular Disease** (ref: no)	2.49 (1.82–3.39) **	2.53 (1.70–3.49) **	2.48 (1.81–3.40) **	2.64 (1.30–5.35) *
**Diabetes** (ref: no)	1.83 (1.36–2.47) **	1.70 (1.23–2.36) *	2.03 (1.54–2.68) **	3.86 (1.43–10.39) *

Note. * *p* < 0.05; ** *p* < 0.01. HR (95% CI) indicates hazard ratios with 95% confidence intervals for the outcome (i.e., mortality). Ref indicates the reference group for each variable for comparison with other groups. For all group comparisons, individuals without depression or elevated CRP were treated as the control group. N indicates the number of individuals for whom the mortality and follow-up data were available.

## Data Availability

The data have not been previously presented orally or by poster at scientific meetings. All of the study data are publicly available at the U.S. Centers for Disease Control and Prevention NHANES website: https://wwwn.cdc.gov/nchs/nhanes/ (accessed on 30 October 2025).
